# Effects of Smartphone-Based Compensatory Cognitive Training and Physical Activity on Cognition, Depression, and Self-Esteem in Women with Subjective Cognitive Decline

**DOI:** 10.3390/brainsci11081029

**Published:** 2021-08-01

**Authors:** Yanghee Pang, Oksoo Kim

**Affiliations:** College of Nursing, Ewha Womans University, Seoul 03760, Korea; anais0220@hanmail.net

**Keywords:** subjective cognitive decline, smartphone, calendar training, walking exercise, depression, self-esteem, nonpharmacologic intervention

## Abstract

Subjective cognitive decline is a symptom that may appear in the early stages of Alzheimer’s disease. This study examined the effects of smartphone-based calendar training and walking exercise regimen on postmenopausal women experiencing subjective cognitive decline. Experimental group 1 participated in both calendar training and walking exercise, group 2 participated in calendar training only, and the control group did not receive either intervention. Forty-two participants completed a cognitive function test and questionnaire upon entering the study and 12 weeks later. The controlled oral word association score increased in experimental groups 1 and 2 and decreased in the control group. Memory contentment increased in experimental group 1, maintained in experimental group 2, and decreased in the control group. Smartphone-based calendar training and a walking exercise regimen improved executive function and memory contentment in everyday life, but the effects on depressive symptoms and self-esteem were not significant. Our findings demonstrate that smartphone-based calendar training and walking exercise improved cognitive function and have potential as nonpharmacologic interventions to strengthen cognitive function in women experiencing subjective cognitive decline.

## 1. Introduction

Subjective cognitive decline (SCD) is defined as the recognition a self-reported decreased in cognitive function even when the individual’s objective cognitive function test results are normal, and is considered the earliest preclinical indicator of Alzheimer’s disease (AD) [1]. SCD may be associated with medical conditions, such as diabetes and hypertension, and emotional problems, such as depression [2], and was associated with frailty in the elderly [3]. Cognitive function in people with SCD is normal, but amyloidosis and neurodegeneration, which are biomarkers of AD, can be observed in the cerebral spinal fluid and magnetic resonance imaging tests [4]. In a previous longitudinal study, the incidence of mild cognitive impairment was 4.9% in elderly individuals without SCD and 18.9% in elderly individuals with SCD, indicating that SCD predicts cognitive impairment [5]. Since SCD can be the earliest preclinical indicator of Alzheimer’s disease, efforts to maintain brain health are needed from the early stages of SCD.

In the United States, 11.2% of adults over the age of 45 have SCD [6]. In China, 14.4–18.8% of individuals aged 60–80 experience SCD, and women (17.4%) were more likely to experience SCD than men (10.5%) [7]. The prevalence of Alzheimer’s disease in Europe was also higher in women (7.2%) than in men (3.4%) [8]. Armeni et al. [9]. reported that middle-aged women with low levels of endogenous sex hormones had lower scores on memory tests. Decreases in estradiol can lead to decreases in synaptic density, affecting the sending and receiving of signals to and from cranial nerve cells, ultimately leading to declines in female cognitive function [10]. Since women experiencing menopause may have increased depression and decreased cognitive function [11], interventions to prevent cognitive decline and reduce depression in menopausal women are needed.

SCD is closely associated with depression [12,13]. Although SCD can be experienced in the preclinical AD stage, it is also associated with psychological states such as depression and anxiety [14]. In people with high levels of depression, neurological changes such as amyloid burden as found in AD are observed [12]. Cognitive decline is correlated with depressive symptoms, and the more the subject felt that they were experiencing cognitive decline, the more sever their depressive symptoms were [15]. In addition, since cognitive impairment is associated with the deterioration of positive psychology constructs such as self-esteem, interventions to evaluate and reinforce self-esteem are needed [16].

In a meta-analysis, nonpharmacologic interventions that increase cognitive and physical activity in subjects with SCD were effective for improving cognitive function, depressive symptoms, and quality of life [17]. Cognitive interventions for people with mild cognitive impairment include compensatory cognitive training, which applies various strategies to reduce functional compromise in daily life, and lifestyle intervention, which improves habits such as regular physical exercise, and healthy nutrition [18].

Compensatory cognitive training can help individuals remember tasks and activities in their daily lives through the use of exeternal memory aids such as alarms, timers, and calendars. Compensatory calendar training, a memory support intervention for individuals with mild cognitive impairment, improves daily life functions and quality of life [19]. Exercise has been shown to be effective as a lifestyle intervention for improving cognitive functions such as memory and executive function [20]. There are mechanisms that explain the effects of exercise on cognitive function [21,22,23,24]. Exercise increases cerebral blood flow and oxygenation to the brain tissue [21]. The brain-derived neurotrophic factor (BDNF) is crucial for brain plasticity and regulation of memory function [22]. Acute exercise increases BDNF concentrations in the peripheral blood of healthy adults [23]. Walking exercise increased the size of the hippocampus and improved memory in the elderly [24]. In addition, walking exercise reduced self-reported cognitive failure and improved subjective cognitive function in breast cancer patients [25]. These findings indicate that the application of nonpharmacologic interventions such as compensatory calendar training and walking-based exercise regimens can improve cognitive function and help maintain daily life functions.

Recently, the effects of virtual reality and web-based intervention studies have been evaluated for improving cognitive function [26,27]. Web-based intervention was effective for improving global and subjective cognition by increasing cognitive and physical activity through computer or smartphone applications [27]. Smartphone-based memory training that presented tasks of attention, memory and working memory improved memory and executive function among older adults with normal cognition or subjective cognitive decline [28,29]. Physical activity intervention using smartphone applications were effective in increasing physical activity [30,31]. The daily step count significantly increased in those who performed walking exercises in conjunction with the smartphone application compared to those who did not [32]. In Korea, the penetration rate of smartphones across all age groups is 87.2% [33], and since smartphones can be used by individuals at convenient times and in comfortable settings, smartphone-based interventions can be effective for cognitive improvement.

Individuals with SCD represent important targets of intervention. For individuals who have preclinical AD, prevention-intervention could slow the rate of incipient decline, thus prolonging and preserving cognitive and functional abilities [2]. The hypothesis of this study was that compensatory cognitive training could improve cognitive function, reduce depression, and increase self-esteem in women with SCD, and that the effect would be greater if accompanied by walking exercise. Thus, the purpose of this study was to identify the effects of smartphone-based compensatory cognitive training and physical activity in menopausal women experiencing SCD.

## 2. Materials and Methods

### 2.1. Study Design

We employed a quasi-experimental, non-equivalent control group, non-synchronized design in this study. Experimental group 1 participated in both calendar training and exercise, group 2 participated in calendar training only, and the control group did not receive either intervention. We asked the participants to complete cognitive function tests and questionnaires upon entering the study and 12 weeks later to assess their cognitive function, depressive symptoms, and self-esteem. This study was conducted with approval from the Institutional Review Board (ewha-201907-0008-02) at Ewha Womans University, Seoul.

### 2.2. Participants

Participants were recruited from five churches and one welfare center for the elderly in metropolitan areas. The inclusion criteria were: (a) menopausal women over 50 years of age, (b) a Mini Mental State Examination-2 [34] score above 26 points indicating normal cognitive function, and (c) a Subjective Cognitive Questionnaire [35] score above 7 points indicating self-perceived cognitive decline. Individuals who were diagnosed with depression or participating in regular exercise (825 MET-minute/weeks) over the prior month were excluded.

The sample size was calculated using the G*Power 3.1.9.2 program based on a significance level of *p* < 0.05, power of 0.8, and effect size of 0.5, following Smart et al. [17]. We applied repeated measurements of multivariate analysis of variance (MANOVA) for analysis, and the appropriate sample size was determined to be 42. Each experimental group therefore required at least 14 participants. We recruited 18 participants for each group to compensate for possible dropouts.

**Figure 1:**
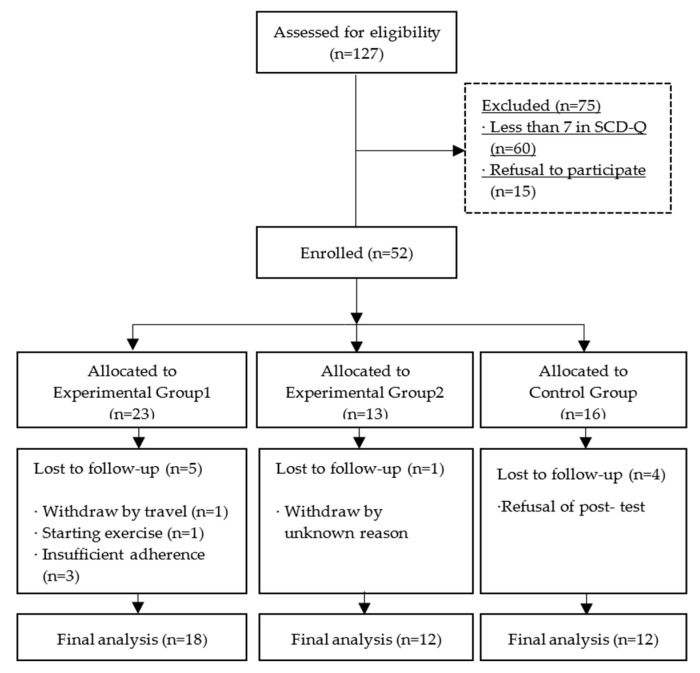
Flow chart of participants.

A total of 127 participants were recruited, and among them, 75 participants were excluded due to SCD-Q scores lower than 7 points (60 participants) and refusal to participate (15 participants). Participants recruited from August to October 2019 were assigned to experimental group 1, those recruited from November to December 2019 were assigned to experimental group 2, and those recruited from January to February 2020 were assigned to the control group. It was confirmed that participants in experimental group 2 and control group did not engage in regular walking exercise during the intervention study period. In the final analysis, 5 participants from experimental group 1, 1 from experimental group 2, and 4 from the control group were excluded. The reasons for dropout are shown in Figure 1. In conclusion, 42 participants were analyzed.

### 2.3. Interventions

A calendar application was used to improve memory regarding daily life appointments and tasks. A health application was used to manage the walking-based exercise regimen. This intervention was designed to enable individual participation at each subject’s leisure. The intervention period was 12 weeks. Up to 4 weeks after the commencement of the intervention, orientation, application usage training, and confirmation of adherence were conducted through face-to-face meetings; text messages were sent once per week to encourage implementation during weeks 5 to 7 and weeks 9 to 11 without face-to-meetings with the researcher. At 8 weeks and 12 weeks, a researcher met with participants to confirm their adherence to the intervention.

#### 2.3.1. Calendar Training

Calendar training consisted of the following three sections: (1) appointments, (2) items to be completed, and (3) journaling [36]. In the appointment section, participants recorded tasks that needed to be performed at specific times. In the items to be completed section, participants created a to-do list without indicating when the items were to be completed. In the journaling section, participants wrote about important events that happened to them that day. We used the One Day calendar application (Yuna Soft, Inc, Daejeon, Korea) to implement calendar training and distributed the application free of charge.

#### 2.3.2. Walking Exercise

The walking exercise intervention was self-managed. The daily target number of walking steps were determined by the participants themselves within the recommended 6000–8500 steps range [37] for adults aged 50 or older without chronic diseases. For participants with chronic diseases, the target numbers of steps were based on the recommended standard of 5000 steps for heart failure patients [38]. Walking exercise was recommended for at least 60 min a day at moderate intensity. The health application we used to set daily target steps was produced by S Electronics and provided free of charge. The health application automatically sends feedback regarding the achievement of daily step targets through smartphone notifications, and the degree of achievement and number of steps are automatically recorded and saved.

### 2.4. Data Collection and Outcome measures

Prior to the commencement of intervention, we obtained consent for participation in the study and administered questionnaires to assess memory contentment, depressive symptoms, and self-esteem. Trained research assistants administered the Seoul Neuropsychological Battery-Core (SNSB-C) [39] individually in quiet settings to assess cognitive function.

#### 2.4.1. Cognitive Function

Cognitive function was measured by the attention, memory, and executive function test items on SNSB-C. Attention was assessed using the Digit Span Test-Forward (DST-F) and Digit Span Test-Backward (DST-B). The possible score ranges of DST-F and DST-B are 0–9 and 0–8, respectively. Higher scores indicate better attention.

Memory was assessed with the Seoul Verbal Learning Test-Elderly version (SVLT-E). The SVLT-E consists of immediate recall, delayed recall, and recognition. In this study, memory was evaluated using immediate and delayed recall tests. The score ranges from 0–36 points for immediate recall and 0–12 points for delayed recall, with higher scores indicating better language memory.

Executive function was assessed with the Korean-Color Word Stroop Test-60 Seconds (K-CWST-60) and Controlled Oral Word Association Test (COWAT). The scores range from 0–112 points for K-CWST-60, with higher scores indicating better executive function. The COWAT consists of category fluency and word fluency. Each score ranges from 0–30 and the combined score range is 0–60, with higher scores indicating better executive function.

Memory contentment was measured using the Multifactorial Memory Questionnaire-Contentment (MMQ-C) [40]. The MMQ-C contains 18 items addressing a variety of perceptions that participants may have about their current memory ability. Each item is scored on a five-point Likert scale with scoring as follows: 0 (strongly disagree); 1 (disagree); 2 (undecided); 3 (agree); and 4 (strongly agree). Total scores range from 0–72, with higher scores indicating great contentment. Cronbach’s alpha in this study was 0.91.

#### 2.4.2. Depressive Symptoms

Depressive symptoms were measured using the Geriatric Depression Scale (GDS) [41]. In this study, we used a Korean version of GDS [42]. The GDS contains 30 items concerning feelings or thoughts over the prior week. Each item response is either yes or no and the possible score ranges from 0 to 30 with a cut-off point of 18. Cronbach’s alpha in this study was 0.87.

#### 2.4.3. Self-Esteem

Self-esteem was measured with the Rosenberg’s Self-Esteem Scale (SES). This tool contains 10 items and is scored on a five-point Likert scale: 1 (strongly disagree); 2 (disagree); 3 (undecided); 4 (agree); 5 (strongly agree). Total scores range from 10–50, with higher scores indicating higher self-esteem. Cronbach’s alpha in this study was 0.79.

#### 2.4.4. Adherence

Adherence to calendar training was assessed by selecting a two-day period during the previous week during face-to-face meeting. Compliance was defined as a score of 7 or more points. Criteria from Greenway et al. [36] were modified for use in the smartphone application as follows:

1. 2 points if an appointment, to-do item, or journaling was completed more than 2 days per week;
2. 2 points if there was a record of the appointment;
3. 2 points if there was at least one recorded to-do item;
4. 4 points if there was a record in the journaling area.

Adherence to the walking exercise intervention was assessed to confirm that the walking exercise was carried out at least five times per week with at least 5000 steps a day.

In Experimental Group 1, 3 participants with insufficient walking exercise and calendar writing dropped out, and in Experimental Group 2, 1 participant withdrew from the study.

### 2.5. Data Analysis

Statistical analyses were conducted using SPSS version 26.0. Descriptive statistics, including the mean, standard deviation (SD), frequency, and percentage, were calculated for participant characteristics and main variables. Fisher’s exact test and ANOVA were used to identify differences in characteristics between groups. We performed 2-way repeated measure MANOVA, which set the group and time as independent variables to examine the significance of group, time, and interaction effects on changes in outcome variables. Box’s M test was performed to confirm whether the covariance matrix of outcome variables between groups was homogeneous, and we confirmed that the covariance matrix was not homogeneous. When the sample size is small and the covariance matrix identity is violated, it is necessary to use more stringent statistics [43]. Therefore the significance of the model was determined using Pillai’s T, which is the most stringent standard among the verification statistics for MANOVA.

## 3. Results

### 3.1. Participant Characteristics

The participants’ age ranged from 51 to 75 years, with a mean age of 60 years. Years of education ranged from 9 to 20 years. Demographic characteristics of the participants in each group are shown in Table 1. There were no significant differences in age (*F* = 0.305, *p =* 0.739), years of education (*F =* 0.172, *p =* 0.843), marital status (*χ^2^ =* 8.458, *p =* 0.089), cohabiting family (*χ^2^ =* 9.553, *p =* 0.237), occupational status (*χ^2^* = 2.716, *p* = 0.284), or hobby (*χ^2^*= 1.512, *p* = 0.539) between groups.

### 3.2. Cognitive Function, Depressive Symptoms, and Self-Esteem among Groups at Baseline

The differences in mean score between the groups for cognitive function, depressive symptoms, and self-esteem between groups were analyzed. None of the variables, except the digit span test-backward (DST-B) and controlled oral word association (COWAT), significantly differed between groups. DST-B was significantly different between groups (*F =* 4.925, *p =* 0.019) and experimental group 1 showed a significantly lower mean value than experimental group 2 after post hoc analyses. COWAT scores were also significantly different between groups (*F =* 3.541, *p =* 0.039) with lower scores in experimental group 1. The mean score and differences in outcome variables are presented in Table 2.

### 3.3. Effects of Intervention on Cognitive Function, Depressive Symptoms, and Self-Esteem

**Figure 2:**
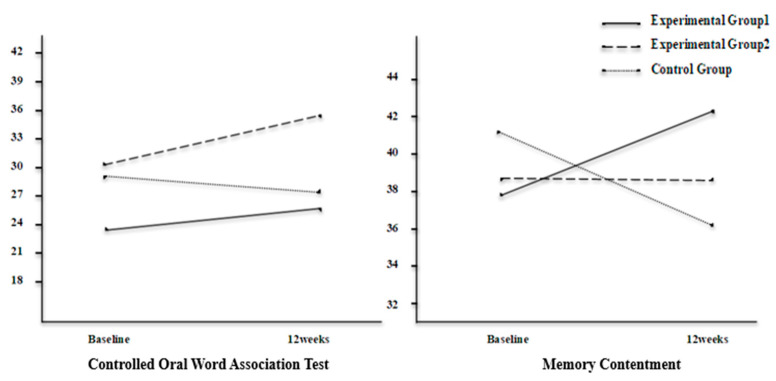
Interaction effects in cognitive function.

The interaction effects of group and time were confirmed with MANOVA and were significant (*Pillai’s T* = 0.677, *p* = 0.042). The changes in scores of cognitive function, depressive symptoms, and self-esteem for each group are presented in Table 3. The interaction effects of group and time showed that the significant variables were COWAT (*F* = 4.869, *p* = 0.013) and memory contentment (*F* = 4.789, *p* = 0.014) which means that the effectiveness varied between groups over time. The COWAT scores showed a tendency to increase in experimental groups 1 and 2 and to decrease in the control group after 12 weeks. Memory contentment showed a tendency to increase in experimental group 1, to remain steady in experimental group 2, and to decrease in the control group after 12 weeks. Significant interaction effects were confirmed through a Plot diagram (Figure 2).

## 4. Discussion

In this study, we evaluated the effects of smartphone-based calendar training and a walking exercise intervention on cognitive function, depressive symptoms, and self-esteem in women with SCD. After the intervention, the COWAT score was used for confirming executive function. The COWAT score increased in experimental groups 1 and 2 but decreased in the control group, and the interaction effect was significant. In a previous study of memory improvement training strategies for elderly individuals living in the community, there were significant improvements in COWAT scores [44]. In this study, for compensatory cognitive training, participants were asked to record appointments or to-do lists on a calendar application and write a simple diary. Thus, the COWAT score, which represents verbal fluency, should have improved. However, in a previous study that applied memory support intervention using a calendar among subjects with mild cognitive impairment, objective cognitive functional improvements such as increased verbal fluency were not observed [19,36]. This indicates that applying cognitive interventions during the SCD stage before objective cognitive impairment occurs is more effective for improving cognitive function.

After intervention, memory contentment increased in experimental group 1 and was maintained in experimental group 2, but decreased in the control group. Gokal et al. [25] reported that walking exercise improved subjective evaluations of cognitive function in breast cancer patients receiving chemotherapy. This is consistent with the results of this study, and we concluded that walking exercises improve memory satisfaction.

Walking exercise prescribed to elderly subjects for 6 months increased the size of the hippocampus, which is related to memory [24]. Moderate-intensity aerobic exercise improved cognitive function in adults over 50 [45]. These findings suggest that aerobic exercise, such as walking, helps to prevent degenerative changes in the brain, thus improving memory related to daily life. In terms of effects related to cognitive function, executive function and memory satisfaction were improved in experimental group 1, which received both calendar training and walking interventions. Therefore, we confirmed that applying both cognitive and physical activity interventions is especially effective for improving cognitive function [46].

Depressive symptoms were significantly reduced in experimental group 1, which received both smartphone-based calendar training and walking interventions, but the interaction effects with time and group were not significant. Cognitive decline is closely related to depressive symptoms, and the level of depressive symptoms increases as cognitive function decreases [15]. Participants in this study were not objectively found to have declines in cognitive function, and did not have high levels of depressive symptoms in pretests, therefore the interaction effect might not be significant. However, in experimental group 1, which performed walking exercise, the level of depressive symptoms was significantly lowered. Therefore, walking regimens are thought to prevent and manage depressive symptoms among those with reduced cognitive function.

In this study, we confirmed the effect of smartphone-based calendar training and walking exercise on self-esteem. Self-esteem significantly increased in experimental group 1, where calendar training and walking exercise were prescribed together, but the interaction effect was not significant. In a study that applied self-managed walking exercise interventions in breast cancer patients who received chemotherapy, self-esteem significantly increased after intervention [47]. In this study, as in Gokal et al. [47], self-esteem should have improved, as the participants set their own step targets and applied the walking exercise intervention at their leisure, at comfortable times and places. The interaction effect might not be significant in this study because the participants were capable of going about their daily lives unassisted and their self-esteem was not severely degraded. Cognitive dysfunction is associated with declines in positive psychology constructs such as self-esteem [16]. Therefore, it is necessary to maintain a positive psychological state and prevent cognitive dysfunction through the reinforcement of cognitive and physical activities from middle to old age, when cognitive decline begins to occur.

The subjects of previous studies of cognitive interventions mostly exhibited objective cognitive impairment and were elderly. Most of these studies applied cognitive interventions to participants living in facilities. However, in this study, smartphone-based interventions were individually applied among participants who experienced SCD and lived in the community. Based on the results of this study, nurses could recommend smartphone-based calendar training and walking-based exercise regimens to individuals with subjective cognitive decline or mild cognitive impairment. In this study, the intervention effect was confirmed in experimental group 1, in which calendar training and exercise were applied together, and in experimental group 2, in which only memory support was administered. We suggest that to improve cognitive function of women with SCD, both cognitive and physical activities should be recommended.

In this study, we used a convenience sample drawn from specific religious institutions and elderly welfare institutions located in the metropolitan area of Seoul, so there are limitations in generalizing our findings. It was difficult to recruit all participants at once, and therefore each group was sequentially recruited. The limitations of this study were that the nutritional status and body composition, which could affect the participants’ cognitive function, were not considered, and a group that utilized only exercise was not assigned. Because the number of participants in this study was small, the study results should be interpreted with caution. In the future, repeated studies with a larger number of participants will be needed. The intervention applied in this study had an effect on cognitive function improvement, but not depressive symptoms or self-esteem. Therefore, it is necessary to confirm the effects of these interventions on psychological states in future studies.

## 5. Conclusions

SCD may appear in the early stages of Alzheimer’s disease and develop into cognitive dysfunction, so preventive intervention is required. In this study, convenient smartphone-based interventions were applied among middle-aged and elderly women living in the community and were found to improve executive function and memory contentment in everyday life. Smartphone-based interventions have the advantages of allowing individual participation without restraints on time or location and improving patient adherence to the intervention. Smartphone-based calendar training and walking interventions could be applied to strengthen the cognitive function of women experiencing SCD.

## Figures and Tables

**Table 1:** Demographic characteristics of participants (N = 42).

		Experimental Group 1 (n = 18)	Experimental Group 2 (n = 12)	Control Group (n = 12)	χ^2^/*F*	*p*
		n (%) or M ± SD				
Age (years)		60.89 ± 6.62	59.42 ± 5.16	59.33 ± 6.54	0.305	0.739
Education years		12.94 ± 3.06	13.58 ± 2.94	13.33 ± 2.96	0.172	0.843
Marital status	single	3 (16.7)	0	0	8.458	0.089
	married	10 (55.6)	12 (100.0)	10 (83.3)		
	divorced/ widowed	5 (27.7)	0	2 (16.7)		
Cohabiting family	spouse	3 (16.7)	4 (33.3)	3 (25.0)	9.553	0.237
	children	3 (16.7)	0	1 (8.3)		
	spouse & children	7 (38.9)	8 (66.7)	6 (50.0)		
	living alone	5 (27.7)	0	2 (16.7)		
Occupation	Yes	14 (77.8)	7 (58.3)	6 (50.0)	2.716	0.284
	No	4 (22.2)	5 (41.7)	6 (50.0)		
Hobbies (reading, puzzles, etc.)	Yes	8 (44.4)	8 (66.7)	7 (58.3)	1.512	0.539
	No	10 (55.6)	4 (33.3)	5 (41.7)		

**Table 2:** Cognitive function, depressive symptoms, and self-esteem at baseline (N = 42).

		Experimental Group1 (n = 18)	Experimental Group2 (n = 12)	Control Group (n = 12)	*F*	*p*
		M ± SD				
Attention	DST-F	6.56 ± 1.20	7.67 ± 1.07	6.75 ± 1.60	2.812	0.072
	DST-B	4.11 ± 0.96	5.75 ± 4.60	4.58 ± 1.83	4.925	0.019 *
Memory	Immediate Recall	23.11 ± 3.76	24.00 ± 5.26	23.25 ± 5.36	0.138	0.871
	Delayed Recall	8.56 ± 1.85	8.17 ± 2.25	8.83 ± 2.76	0.266	0.768
Executive function	K-CWST-60	50.22 ± 8.04	56.42 ± 7.22	56.00 ± 10.42	2.515	0.094
	COWAT	23.50 ± 7.20	30.75 ± 7.70	29.50 ± 9.56	3.541	0.039 *
Memory contentment		37.83 ± 7.45	38.75 ± 11.33	41.25 ± 7.98	0.547	0.583
Depressive symptoms		10.50 ± 4.93	13.50 ± 6.68	10.33 ± 5.07	1.328	0.277
Self-esteem		33.44 ± 5.48	35.42 ± 5.45	33.83 ± 3.74	0.578	0.566

* *p* < 0.05.

**Table 3:** Effects of intervention on cognitive function, depressive symptoms, and self-esteem (N = 42).

		Experimental Group 1 (n = 18)		Experimental Group 2 (n = 12)		Control Group (n = 12)		*F* (Time)	*F* (Group)	*F* (Time * Group)
		Pre	Post	Pre	Post	Pre	Post			
Attention	DST-F	6.56 ± 1.20	6.72 ± 1.27	7.67 ± 1.07	7.58 ± 1.44	6.75 ± 1.60	7.42 ± 1.38	1.298	2.773	0.906
	DST-B	4.11 ± 0.96	4.72 ± 1.60	5.75 ± 4.60	6.25 ± 1.14	4.58 ± 1.83	5.17 ± 1.34	3.400	8.464 **	0.012
Memory	Immediate Recall	23.11 ± 3.76	26.94 ± 3.40	24.00 ± 5.26	28.75 ± 4.81	23.25 ± 5.36	26.67 ± 3.92	36.858 ***	0.557	0.322
	Delayed Recall	8.56 ± 1.85	9.67 ± 1.50	8.17 ± 2.25	10.33 ± 2.19	8.83 ± 2.76	10.42 ± 1.83	24.632 ***	0.314	0.932
Executive function	K-CWST-60	50.22 ± 8.04	56.89 ± 11.12	56.42 ± 7.22	65.08 ± 13.62	56.00 ± 10.42	57.75 ± 9.86	18.824 ***	2.174	2.214
	COWAT	23.50 ± 7.20	26.17 ± 7.23	30.75 ± 7.70	36.17 ± 5.00	29.50 ± 9.56	27.67 ± 6.87	5.296 *	5.854 **	4.869 *
Memory contentment		37.83 ± 7.45	42.33 ± 8.56	38.75 ± 11.33	38.67 ± 10.53	41.25 ± 7.98	36.25 ± 9.89	.022	0.140	4.789 *
Depressive symptoms		10.50 ± 4.93	7.89 ± 5.17	13.50 ± 6.68	12.17 ± 7.27	10.33 ± 5.07	11.58 ± 5.57	1.192	1.849	1.969
Self-esteem		33.44 ± 5.48	35.28 ± 5.63	35.42 ± 5.45	34.58 ± 4.60	33.83 ± 3.74	34.33 ± 4.89	0.545	0.126	1.393

* *p* < 0.05, ** *p* < 0.01, *** *p* < 0.001.

## Data Availability

Not applicable.
